# Late Sac Rupture due to a Type IV Endoleak after Previous Endovascular Aortic Aneurysm Repair: A Case Report

**DOI:** 10.3389/fsurg.2017.00045

**Published:** 2017-08-10

**Authors:** Konstantinos Filis, Constantinos Zarmakoupis, Georgios Karantzikos, Frangiska Sigala, Gerasimos Bazigos, George Galyfos

**Affiliations:** ^1^First Department of Propaedeutic Surgery, University of Athens Medical School, Hippocration Hospital, Athens, Greece

**Keywords:** type IV endoleak, rupture, endovascular aortic aneurysm repair, open repair, microleakage

## Abstract

Type IV endoleaks have been identified as endoleaks of low flow, and rupture risk has been estimated to be minimal in literature. Therefore, conservative treatment has been recommended in most cases. We are presenting a rare case of late rupture due to type IV endoleak that was treated with open repair applying a novel surgical technique.

## Introduction

Endoleaks have been identified as the most frequent complications after endovascular aortic aneurysm repair (EVAR), with type II endoleaks comprising the majority (1). Although proper management of type I–III endoleaks has been extensively evaluated in the literature, data on treatment of type IV and V endoleaks are limited (2). Type IV endoleaks have been attributed to the porosity of the graft fabric and, by definition, they are detected within the first postoperative month (3). Although type I–III endoleaks have been associated with abdominal aortic aneurysm rupture and high re-intervention rates (4), type IV endoleaks are considered endoleaks of low flow and their rupture risk is minimal (5). Therefore, the aim of this report is to present a rare case of a late aneurysm rupture due to a type IV endoleak after a previous EVAR procedure.

## Case Report

**Figure 1:**
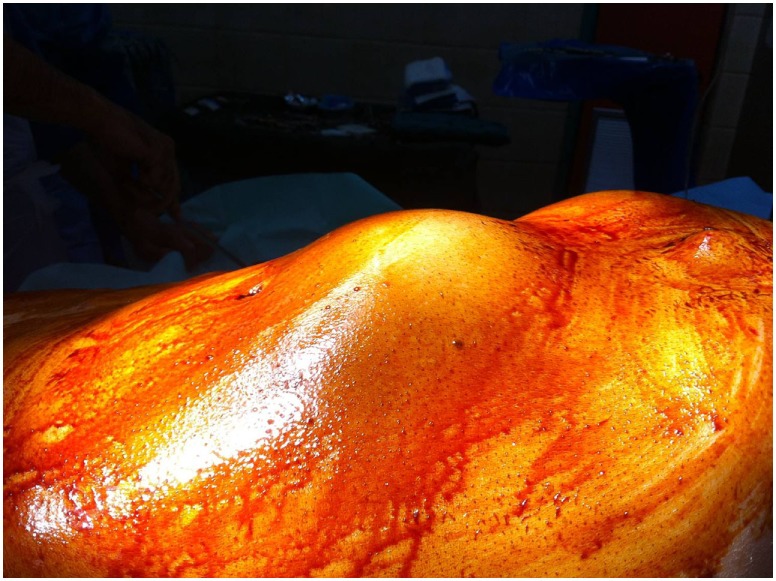
A large pulsatile abdominal mass observed during clinical examination.

A 76-year-old patient was transferred to our emergency department suffering from abdominal and lumbar pain for almost 5 days. Physical examination revealed a large pulsatile abdominal mass (Figure 1), low blood pressure (90/50 mmHg), and an increased heart rate (95/min). His medical history included hypertension, atrial fibrillation, dyslipidemia as well as a prior EVAR procedure conducted almost 9 years ago (original aneurysm size: 5.6 cm in diameter). The previous EVAR was followed closely for almost 3 years without detecting any aneurysm size change or endoleak. Regarding the type of implanted endograft, the patient did not give any information or have any industry documents to prove the type of graft. Therefore, only assumptions could be made by the authors, and they could not report the exact type with certainty. In addition, the patient was receiving antihypertensive drugs, a statin regimen, aspirin, and acenocoumarol. Finally, the laboratory investigation revealed anemia (hematocrit = 28%) as well.

**Figure 2:**
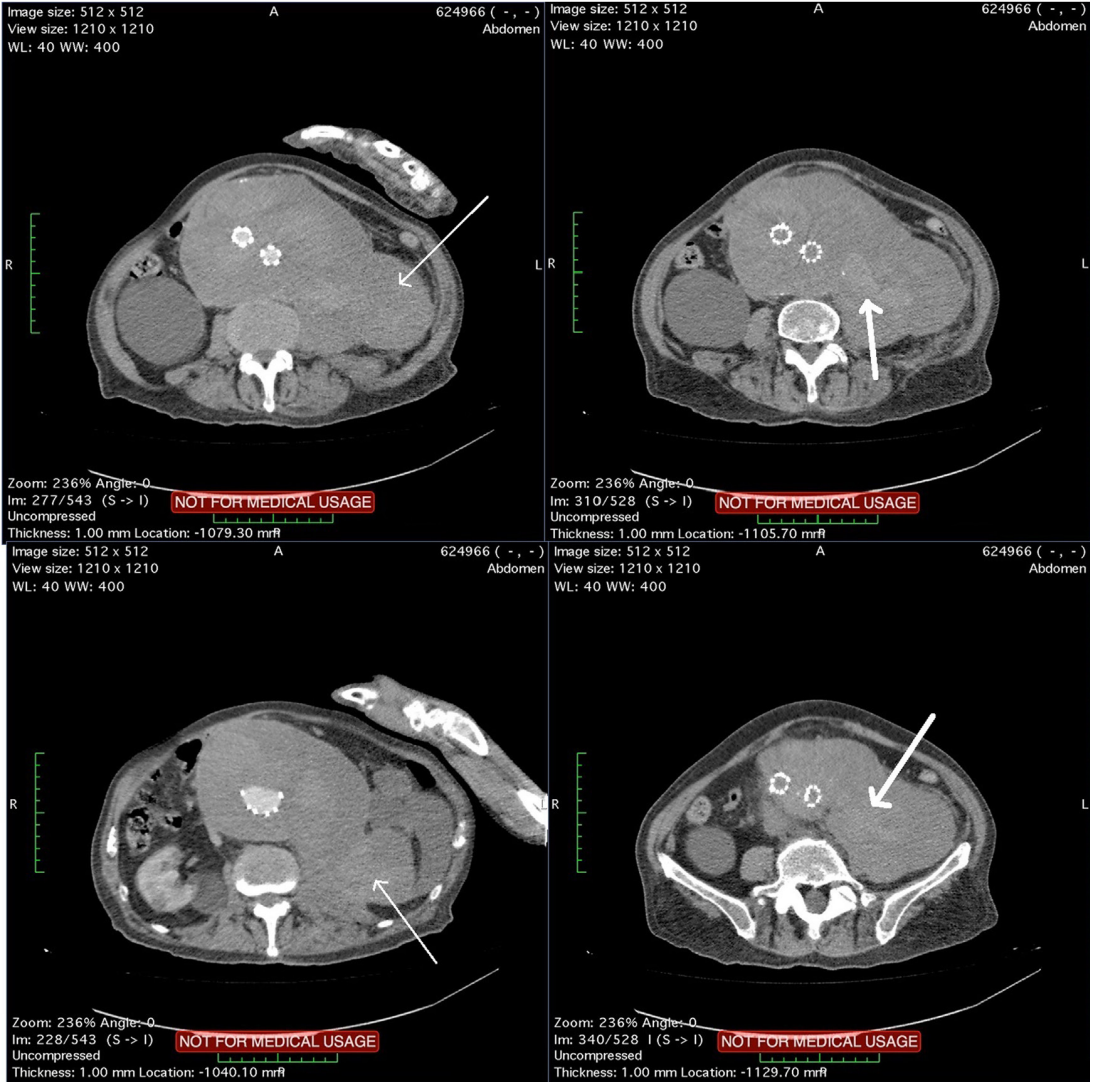
Computed tomography angiography images showing a rupture of the aneurysm sac that causes a large retroperitoneal hematoma (thin arrows). Blood pools are observed within the hematoma (thick arrows) although no typical endoleak can be identified.

**Figure 3:**
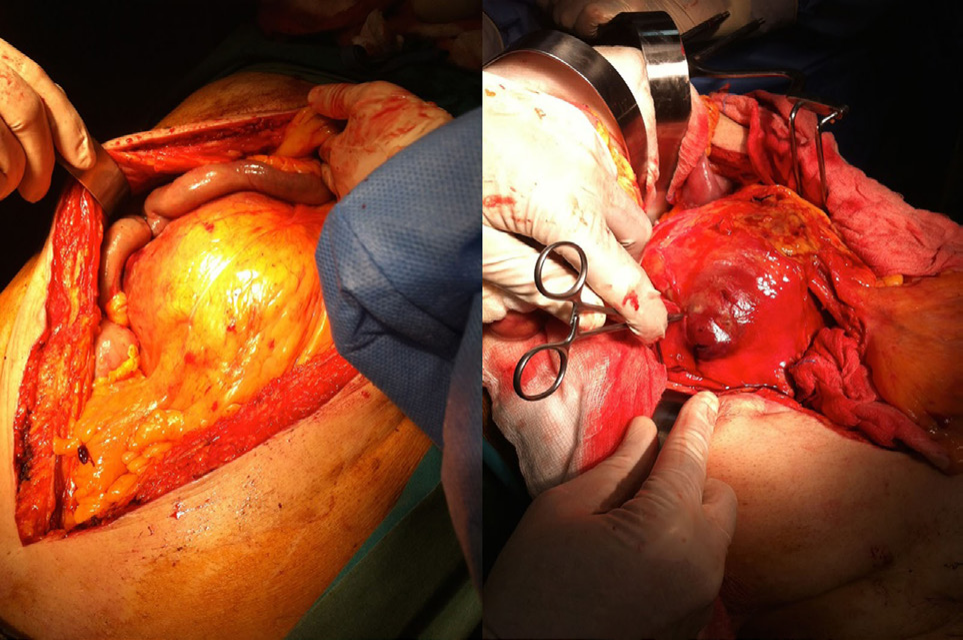
The patient received a laparotomy (left figure) and a large retroperitoneal hematoma was identified (right figure).

**Figure 4:**
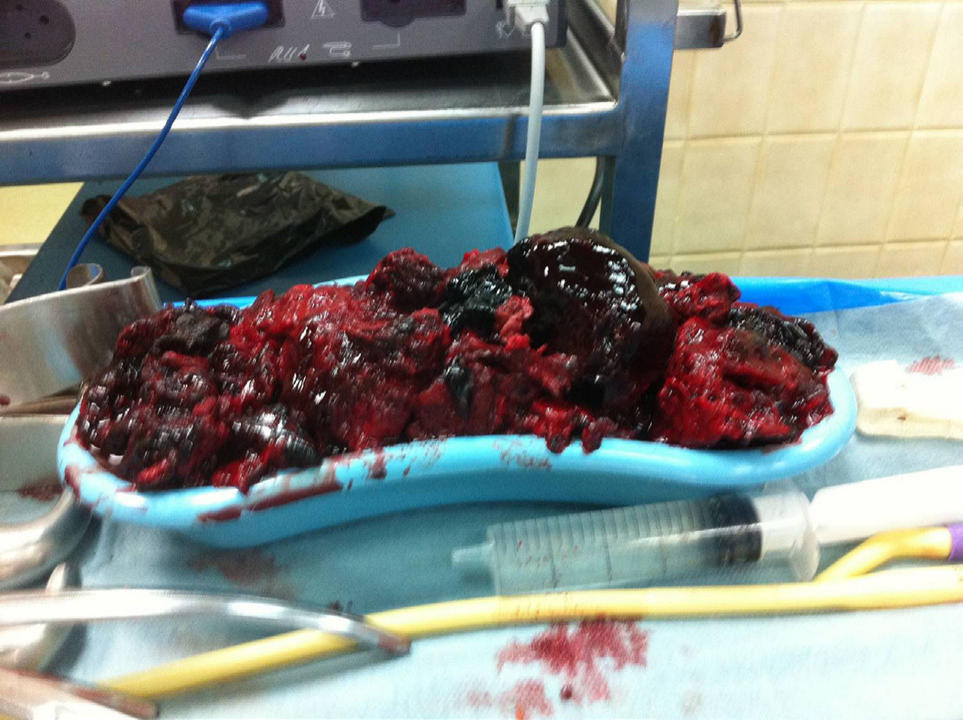
A large amount of thrombus/hematoma removed from within the aneurysm sac.

**Figure 5:**
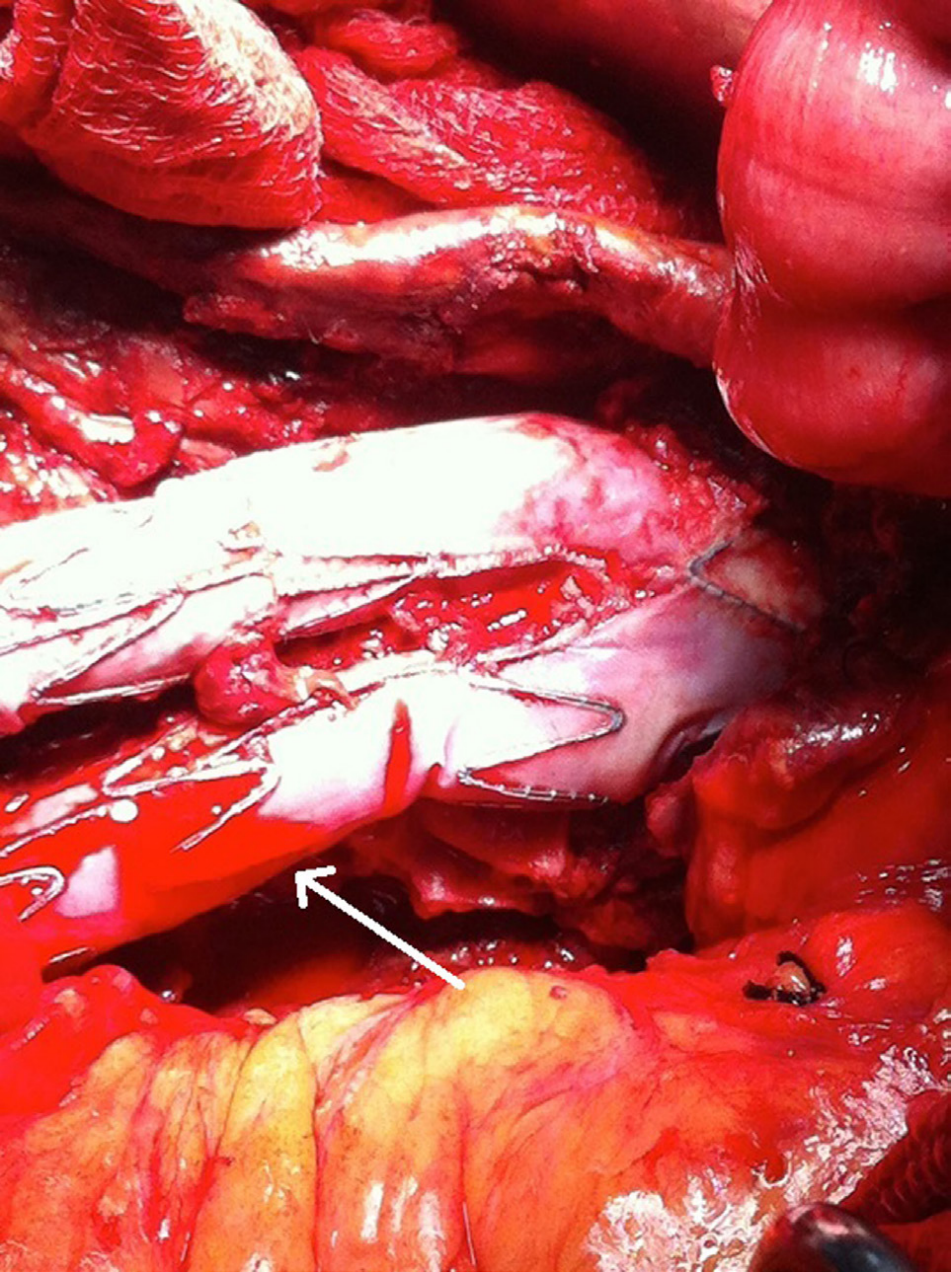
A microleakage identified to originate from the middle part (non-overlapping) of the left graft limb (arrow).

**Figure 6:**
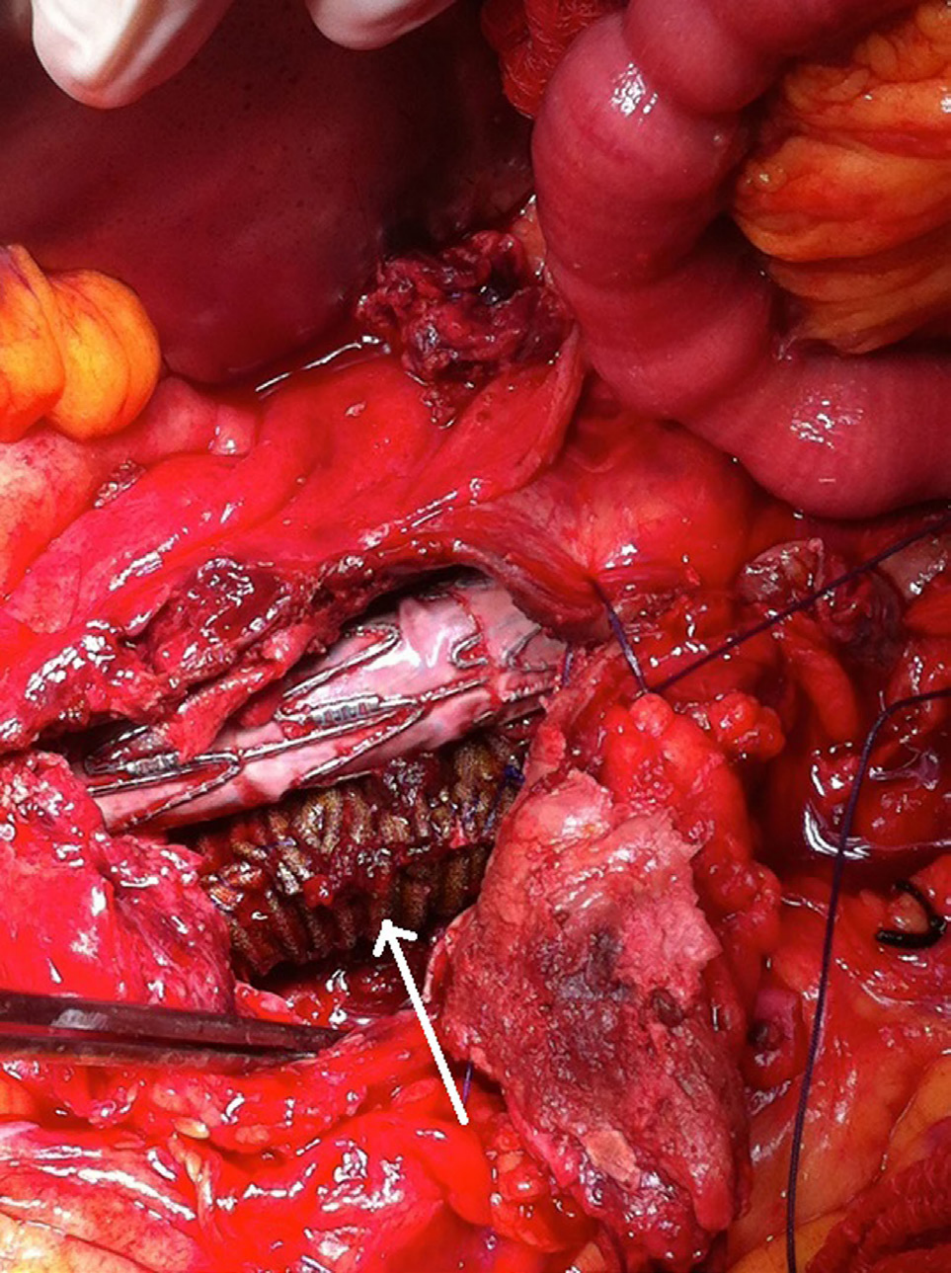
A synthetic silver-coated Dacron patch (arrow) was wrapped around the left graft limb in order to seal the microleakage. The aneurysm sac was sutured to cover the endograft.

**Figure 7:**
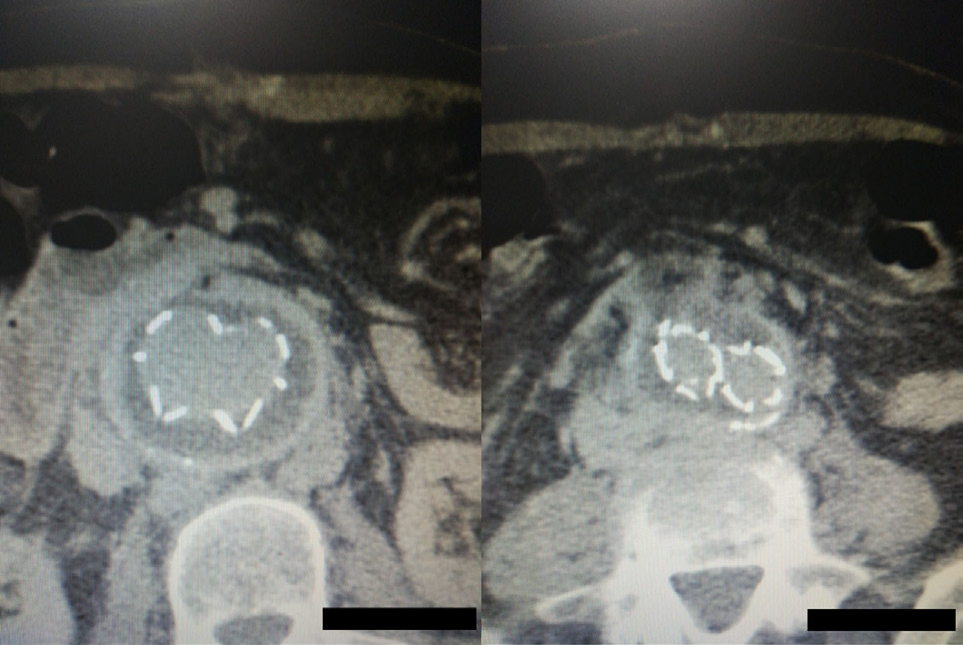
Postoperative computed tomography angiography images showing the graft wrapped by the sac without any sac enlargement or obvious endoleak.

The patient underwent a computed tomography angiography (CTA) that revealed an intact endovascular graft, an enlargement of the aortic aneurysm sac as well as a posterior sac rupture causing a large retroperitoneal hematoma. The CTA images did not show any obvious type of endoleak (Figure 2). The patient underwent an emergency laparotomy where the retroperitoneal hematoma was identified (Figure 3). The aneurysm sac was incised, a large amount of thrombus/hematoma was removed (Figure 4), and leakage was identified through the fabric of the left graft limb (Figure 5), although no patent lumbar artery was found after removal of the thrombus. It was decided by the authors to cover the left limb of the graft with a silver-coated Dacron patch in order to seal the leakage, and the aneurysm sac was sutured over the graft (Figure 6). The patient was transferred to the intensive care unit for postoperative monitoring. The patient was discharged after 2 weeks of hospitalization due to a respiratory tract infection. The post-discharge course of the patient remains uneventful. Postoperative CTA images did not show any enlargement of the sac or any new type of endoleak (Figure 7).

## Discussion

We have described a rare case of late aneurysm rupture due to type IV endoleak that was successfully managed by evacuating the aneurysm sac, wrapping the graft limbs with a synthetic Dacron patch, and suturing the sac over the graft.

Type IV endoleak is defined as an early leakage through the pores of an otherwise intact endovascular graft (6), although some authors have reported cases of such microleakages even 2–3 years after the primary aneurysm repair (7). This type of endoleak has been mainly observed with older versions of endografts although the evolution of grafts has minimized the rate of such endoleaks in the recent years. In addition, such endoleaks are mainly observed around non-overlapping parts of the graft according to literature (7). This concurs with our case where the primary graft was implanted almost a decade before, and the leak was identified originating from the non-overlapping limb of the graft. Furthermore, the pathophysiologic mechanism of such endoleaks seems to be multifactorial. Chronic wear against metallic parts of the graft or external calcified plaques, deformation of the graft caused by balloon dilatation during procedure, or even chronic exposure to pulsatile forces have been implicated to cause such microleakages (7, 8). Finally, such microleakages have been surprisingly associated with continuous aneurysm sac growth during follow-up by some authors (7, 9), although this has not been verified by large multicenter trials (4).

Regarding surveillance and detection, recent meta-analyses have compared all major imaging modalities concerning the detection of endoleaks. Standard CTA seems to prevail in detecting type I and III endoleaks (10) although magnetic resonance imaging (MRI) and contrast-enhanced ultrasound (CEUS) have shown promising results, especially for low-flow endoleaks (11). According to literature, CEUS could also determine the direction of flow even in hypodynamic endoleaks based on their wash-in and wash-out times, showing an advantage over the static CTA images (12, 13). In addition, blood pool contrast agents, used with MRI recently, allow the accumulation of more contrast within the endoleak and without producing excess radiation (14). However, data concerning detection of type IV endoleaks are still lacking, although these endoleaks are typically observed in the immediate post-deployment angiogram. In our case, CTA imaging did not set the diagnosis and only proceeding with an open repair could identify the exact type of endoleak.

Regarding treatment of type IV endoleaks, any repair would be reasonable only when the aneurysm sac grows or when the patient presents with progressive symptoms (15, 16). Such endoleaks have been usually reported in patients under full coagulation treatment—such as in our case—and they seem to spontaneously resolve in the majority of cases when coagulation status is normalized (17). Experimental studies have also reported the injection of biocompatible elastomers into the aneurysm sac, even for type IV endoleaks, with promising results (18). Other authors have presented more interventional approaches. Clemens et al. have described the implantation of a surgeon-modified stent graft in order to reline the previous endograft (19). Moreover, Wachal et al. have reported a late sac increase due to type IV endoleak where they proceeded with open surgery and suturing of the graft defects (20). However, in our case, a silver-coated Dacron patch was used to cover and seal both graft limbs with satisfying outcome.

In conclusion, type IV endoleaks could present even years after primary EVAR and they could even lead to rupture, justifying a closer long-term follow-up such as other types of endoleak. Covering the defected fabric with a synthetic patch and closing the aneurysm sac could be applied when an open treatment is needed.

## Ethics Statement

This study was carried out in accordance with the recommendations of the institutional ethics committee with written informed consent from the patient. The patient gave written informed consent in accordance with the Declaration of Helsinki. The protocol was approved by the institutional ethics committee. We clearly state that a written informed consent was obtained from the participant for the publication of this case report.

## Author Contributions

KF: design and overall responsibility; CZ: design and writing; GK and FS: literature search; GB: writing; and GG: writing and drafting.

## Conflict of Interest Statement

The authors declare that the research was conducted in the absence of any commercial or financial relationships that could be construed as a potential conflict of interest.
